# Pediatricians’ awareness of galenic drugs for children with special needs: a regional survey

**DOI:** 10.1186/s13052-023-01462-3

**Published:** 2023-06-19

**Authors:** Francesca Burlo, Davide Zanon, Paola Minghetti, Valentina Taucar, Giulia Benericetti, Giada Bennati, Egidio Barbi, Lucia De Zen

**Affiliations:** 1 Department of Medicine, Surgery, and Health Sciences, University of Trieste, Trieste, Italy; 2 Pharmacy and Clinical Pharmacology Department, Institute for Maternal and Child Health - IRCCS Burlo Garofolo, Trieste, Italy; 3 Dept. Scienze Farmaceutiche, Università degli Studi di Milano, Milano, Italy; 4 Pediatric Palliative Care and Pain Service, Institute for Maternal and Child Health – IRCCS Burlo Garofolo, Trieste, Italy; 5 Pediatric Department, Institute for Maternal and Child Health - IRCCS Burlo Garofolo, Trieste, Italy

**Keywords:** Children, Medical complexity, Galenic compounds, Pediatricians, Magistral formula

## Abstract

The prevalence of children with medical complexity is increasing, therefore drug formulations must be updated in accordance with their needs. Furthermore, a different drug formulation may be also needed for patients who require a very low dosage which is not easily reachable with those of the industrial products or for those following a ketogenic diet. Galenic (or compounded) drugs have been recently pointed out as effective in treating children. Nonetheless, their knowledge among healthcare providers is limited. We investigated how much did pediatricians know about galenic compounds by a short questionnaire administered to family and hospital pediatricians and pediatric residents in Friuli Venezia Giulia, Italy. We collected answers from 65 family pediatricians (57,5%), 39 hospital pediatricians (36,1%), and 47 pediatric residents (41,2%). Overall, both family and hospital pediatricians substantially know what is a galenic compound and the indications to its use. Of note, most of pediatricians ignore which is the legislation that allows the galenic compounds’ preparation and use, and which is the correct procedure to prescribe them. Moreover, half of the hospital pediatricians and one-third of the family ones erroneously stated that galenic formulations cost more or like the industrial products, and around the 15% of both categories affirmed that galenic compounds are less safe than the commercial product. In conclusion, the use of galenic drug may significantly improve children’s and caregivers’ quality of life. We believe that all pediatricians should be updated on this quite new and interesting topic.

## Main text

**Fig. 1:**
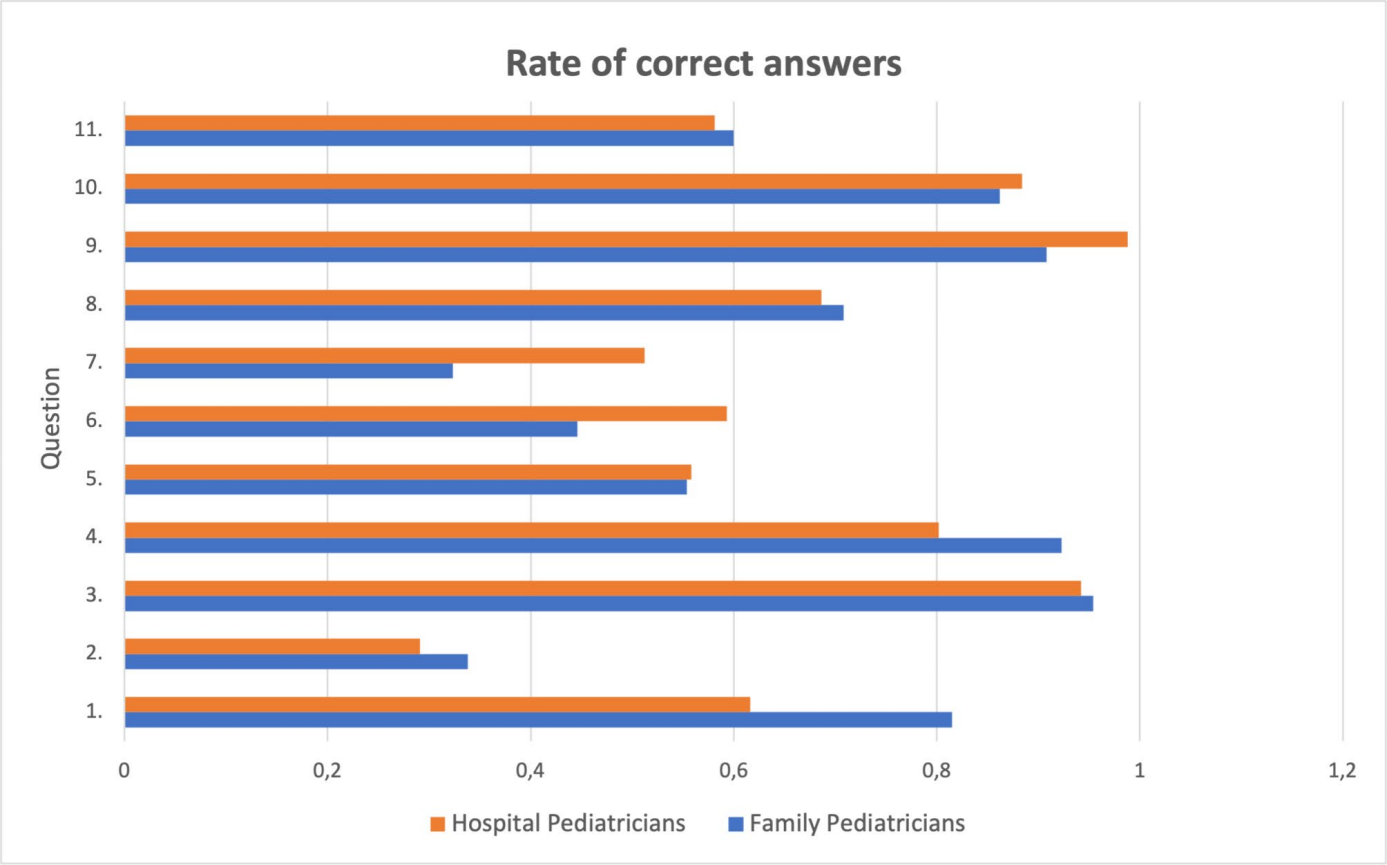
Rate of correct answers

Dear Editor,

The prevalence of children with medical complexity (CMC) is constantly increasing, thanks to the consistent improvements in either survival rates at birth or high-complexity cares [1]. Likewise, the therapeutic approach, and in particular drugs formulations, must be revised and updated in accordance with these children’s needs. CMC are often fed by percutaneous endoscopic gastrostomy (PEG) or nasogastric tube (NGT) because of their severe dysphagia and often consistent sialorrhea. Therefore, specific drug formulations are needed in this setting [2–5]. Caregivers spend a relevant amount of time every day caring for their children and administering therapies [6, 7], and they often have to manipulate drugs (e.g. grinding). By this way, the precise dosage of drug administered is often poorly reliable, and the complexity of the home therapy management may lead to either errors or a poor compliance. Moreover, caregivers often face problems in dealing with the feeding device, such as pushing the drug through the tube, with the consequent risk of obstruction. Besides CMC, a different drug formulation than those available on the market may be needed also for other patients, such as young children who require a too low dosage which is not easily reachable with those of the industrial products, or those children following a ketogenic diet that require sugar-free therapies. Galenic compounds (or magistral formula) have been recently pointed out as effective in treating children, and a promising strategy to simplify the home therapy management of CMC [8–12]. Galenic compounds are drugs prepared in a pharmacy in accordance with a medical prescription. They are safe, convenient to administer and also less expensive than industrial products [8]. Nonetheless, their knowledge among healthcare providers is low, particularly in those areas where the use of these compounds is infrequent. For this reason, we investigated how much did pediatricians know about galenic compounds: definition, legislation that allows their use, indications, main advantages, and differences with the commercial product. An online and anonymous questionnaire with mainly multiple-choice questions was administered to all family pediatricians, hospital pediatricians and pediatric residents working in Friuli Venezia Giulia, Italy (Table 1). In this region, the overall population is of 1,500,000 people, there are two university teaching hospitals, and six other pediatric departments in smaller hospitals. Family pediatricians are guaranteed to all children by the National Health System without any cost for the family, and are homogeneously distributed among the region. Paediatric residents come from the two university pediatric residency schools in the region. All answers were collected and analyzed, to estimate the general knowledge on galenic preparations among pediatricians, and to evaluate whether there were significant differences between family pediatricians and hospital ones. Overall, the questionnaire was administered to 113 family pediatricians, 108 hospital pediatricians, and 114 pediatric residents. We collected answers from 65 family pediatricians (57,5%), 39 hospital pediatricians (36,1%), and 47 pediatric residents (41,2%). The answers of hospital pediatricians and pediatric residents were considered as a whole, therefore from now on we will refer to both pediatric specialists and residents as hospital pediatricians. All answers are shown in Table 1; Fig. 1. Overall, we can state that both family and hospital pediatricians substantially know what is a galenic compound, which are the main indications to its use, and they acknowledged that the use of galenic preparations may improve the caregivers’ compliance. Of note, most of pediatricians ignore which is the legislation that allows the galenic compounds’ preparation and use, and which is the correct procedure to prescribe them. Quite surprisingly, half of the hospital pediatricians and one-third of the family ones erroneously stated that galenic formulations cost more or like the industrial products. Moreover, around the 15% of both categories affirmed that galenic compounds are less safe than the commercial product, mainly because of the lack of supervision and of a constant pharmacists’ training.

Nearly the 60% of either family or hospital pediatricians cared for children who were treated with galenic preparations. Almost all of them stated that their use has improved the caregivers’ home therapy management. Finally, we asked whether there was any drug that they would have liked to be available as galenic formulations. Pediatricians gave various answers, mainly related to antibiotics (e.g. clindamycin, fosfomycin at pediatric formulation), antiepileptics (e.g. endorectal diazepam), proton pump inhibitors, and immunosoppressors (e.g. topic sirolimus).

Some discrepancies between family and hospital pediatricians were found in the answers to four questions. Family pediatricians seemed to be slightly more aware of the definition of galenic preparations (81.5% vs. 61.6%; p 0.008) and of who can prescribe them (92.3% vs. 80.2%; p 0.04). On the other hand, hospital pediatricians seemed to know better which drugs can be prepared as galenic compounds (59.3% vs. 44.6%; p 0.07). This is probably because hospital pediatricians are more aware of the use and prescription of off-label drugs [13], therefore they probably assumed that these medications may be also prescribed as galenic formulations. Finally, hospital pediatricians gave more correct answers about the cost of galenic compounds in relation to the commercial product (51.2% vs. 32.3%; p 0.02).

Moreover, we investigated the role of working experience in the correctness of the answers, by comparing residents’ and pediatricians’ answers. While residents were more aware of which drugs may be prepared as galenic compounds (72.3% vs. 43.6%; p 0.022), specialists were more aware of what are galenic compounds (74.3% vs. 51.1%, p 0.027) and which is the law that defines their use (48.7% vs. 12.8%; p 0.00026). Moreover, obviously specialists cared more for children treated with galenic compounds (76.9% vs. 42.6%; p 0.00019). Finally, specialists seemed to know better how to correctly prescribe galenic compounds (69.2% vs. 44.7%; p 0.022), but as stated above, most of them were not able to describe the correct procedure. Therefore, we can state that, overall, a longer working experience did not consistently contribute to the correctness of the answers.

This survey has limits, mainly related to the low number of answers. Possibly, some pediatricians did not purposely take part to this survey because of the general poor knowledge on this topic. Moreover, we did not investigate gender, age, and working experience of all doctors who took part in this study searching for possible differences in the answers. The point of strength is that, to our knowledge, this is the first study on the topic in the international literature. Anyway, from the answers collected we could draw interesting conclusions which may pave the way to future projects.

The use of galenic drugs may significantly improve children’s and caregivers’ quality of life. We believe that all pediatricians should be updated on this quite new and interesting topic.

**Table 1:** questionnaire

Question:	Correct answer:
1. Do you know what is a galenic compound?	A drug prepared by a pharmacist, following a medical prescription, for a specific patient.
2. Which law defines the use of galenic compounds?	N. 94/1998.
3. When can a galenic compound be prescribed?	When there is a need of a form, dosage, or formulation different from the industrial product’s ones.
4. Who can prescribe a galenic preparation?	All the answers are correct (every hospital doctor, family pediatrician, doctors working in a third-level hospital).
5. Do you know which is the correct procedure to prescribe a galenic compound? If yes, describe it.	/
6. What can include a galenic preparation?	An active substance described in the Pharmacopoeias of the EU Countries or authorized for an off-label use according to the law n. 648/1996.
7. How is the cost of a galenic compound in relation to the industrial product?	Inferior
8. Which is the added value of galenic compounds?	The lack on the market of an analogous form, particularly when drugs are administered through feeding devices (e.g. PEG, NGT).
9. Can the use of galenic compounds improve the caregiver’s compliance? Why?	Yes
10. Do galenic compounds have the same safety profile as the industrial product? Why?	Yes
11. Do you care for patients who are treated with galenic compounds? Do them improve or worsen the home therapy management?	/
12. Are there any drug that you would like to be available as galenic preparations?	/

EU: European Union; PEG: percutaneous endoscopic gastrostomy; NGT: nasogastric tube

The text of the questions is reported in Table 1.

## Data Availability

The datasets used and/or analysed during the current study are available from the corresponding author on reasonable request.

## List of abbreviations

CMC: Children With Medical Complexity
PEG: Percutaneous Endoscopic Gastrostomy
NGT: Nasogastric Tube

## References

[1] Cohen E, Kuo DZ, Agrawal R, Berry JG, Bhagat SK, Simon TD (2011). Children with medical complexity: an emerging population for clinical and research initiatives. Pediatrics.

[2] Esophageal Atresia and Beckwith-Wiedemann Syndrome in one of the Naturally Conceived Discordant Newborn Twins: first report. G. Serra, V. Antona, M. Schierz, D. Vecchio, E. Piro, G. Corsello. Clinical Case Reports. 2018 Jan 13;6(2):399–401. 10.1002/ccr3.1103 PMC5799623 29445485

[3] Novel missense mutation of the TP63 gene in a newborn with Hay-Wells/Ankyloblepharon-Ectodermal Defects-Cleft Lip/Palate (AEC) syndrome: clinical report and follow-up. Gregorio Serra, Vincenzo Antona, Mario Giuffré, Federica Li Pomi, Lucia Lo Scalzo, Ettore Piro, Ingrid Anne Mandy Schierz, Giovanni Corsello. Ital J Pediatr 2021 Sep;47:196. 10.1186/s13052-021-01152-y PMC8479907 34583755

[4] Neonatal hyperinsulinemic hypoglycemia: case report of kabuki syndrome due to a novel KMT2D splicing-site mutation. E. Piro, I.A.M. Schierz, V. Antona, M.P. Pappalardo, M. Giuffrè, G. Serra, G. Corsello. Ital J Pediatr (2020) 46:136. 10.1186/s13052-020-00902-8 PMC7499940 32948218

[5] Novel LRPPRC compound heterozygous mutation in a child with early-onset Leigh syndrome French-Canadian type: case report of an Italian patient. E. Piro, G. Serra, V. Antona, M. Giuffrè, E. Giorgio, F. Sirchia, I.A.M. Schierz, A. Brusco, G. Corsello. Ital J Pediatr (2020) 46:140. 10.1186/s13052-020-00903-7 PMC7517646 32972427

[6] Lazzarin P, Schiavon B, Brugnaro L, Benini F (2018). Parents spend an average of nine hours a day providing palliative care for children at home and need to maintain an average of five life-saving devices. Acta Paediatr.

[7] Abebe E, Scanlon MC, Lee KJ, Chui MA. What do family caregivers do when managing medications for their children with medical complexity? Appl Ergon. 2020 Sep;87:103108. 10.1016/j.apergo.2020.103108 32501256

[8] Zanon D, Burlo F, Scaramuzza G, Maximova N, Matarazzo L, Maggiore G (2022). Safety and Effectiveness of Compounded Galenic Cholic Acid for bile acid synthesis disorder: a Case Report. Endocr Metab Immune Disord Drug Targets.

[9] Zanon D, Tumminelli C, Galimberti AMC, Torelli L, Maestro A, Barbi E (2021). Compounded glycopyrrolate is a compelling choice for drooling children: five years of facility experience. Ital J Pediatr.

[10] Rehn C, Odouard E, Poncet F, Cochat P, Breant V, Dode X (2018). Facteurs influençant l’acceptabilité des formulations galéniques en pédiatrie – revue de la littérature [Factors influencing the acceptability of pediatric galenic formulations]. Ann Pharm Fr.

[11] Zoellner Y, Balp MM, Marco AG (2011). The role of galenic innovation in improving treatment compliance and persistence: three case studies. Clinicoecon Outcomes Res.

[12] Burlo F, Zanon D, Passone E, Toniutti M, Ponis G, Barbi E (2023). Impact of compounded drugs on the caregivers’ burden of home therapy management in pediatric palliative care: a descriptive study. Palliat Med.

[13] De Zen L, Marchetti F, Barbi E, Benini F (2018). Off-label drugs use in pediatric palliative care. Ital J Pediatr.

